# Mycotoxins in Farm Animals: Health Disorders and Preventive Strategies

**DOI:** 10.3390/toxins18090369

**Published:** 2026-08-27

**Authors:** Vasileios G. Papatsiros

**Affiliations:** Clinic of Medicine, Faculty of Veterinary Medicine, University of Thessaly, 43100 Karditsa, Greece; vpapatsiros@vet.uth.gr

Mycotoxin contamination of feedstuffs remains one of the most persistent and economically damaging challenges confronting global animal production. Produced by fungal genera such as *Aspergillus*, *Fusarium*, *Penicillium*, and *Claviceps* under favorable temperature and moisture conditions, mycotoxins compromise the health, welfare, and productivity of farm animals and threaten the safety of animal-derived food products destined for human consumption. Recent global surveys indicate that 60–80% of harvested crops carry detectable mycotoxin residues [1], and multi-mycotoxin co-contamination—rather than exposure to a single toxin—has become increasingly common in commercial feed [2,3]. The resulting economic burden is substantial: recent estimates place combined food- and feed-chain losses from mycotoxin contamination at several billion US dollars annually worldwide, driven by trade disruption, regulatory enforcement costs, and reduced feed-conversion efficiency across livestock and aquaculture production [4]. It is against this backdrop that the Special Issue “Mycotoxins in Farm Animals: Health Disorders and Preventive Strategies” was launched, to bring together original research addressing the clinical, pathophysiological, and preventive dimensions of mycotoxicoses in livestock and poultry. Eight original research articles were ultimately published in this Special Issue, together offering one of the more comprehensive single-collection perspectives to date on how mycotoxin exposure is detected, mitigated, and managed across production species. This Editorial reflects on their collective contribution and situates them within the broader trajectory of the field.

## 1. Overview of the Special Issue Contributions

The contributions to this Special Issue can be grouped into three complementary themes: (i) mitigation and detoxification strategies, (ii) species- and toxin-specific pathophysiology, and (iii) environmental surveillance and exposure pathways (Table 1).

A substantial share of the Special Issue was devoted to evaluating practical mitigation strategies against mycotoxin exposure in production animals. Papatsiros et al. (contribution 1) demonstrated that a clay-based, multicomponent detoxifying agent alleviated oxidative stress and improved productive performance in sows, while Raj et al. (contribution 2) extended this line of investigation to weaned piglets under combined dietary exposure to deoxynivalenol (DON) and zearalenone (ZEN), reporting improved growth performance and reduced systemic mycotoxin absorption. Complementing these adsorbent-based approaches, Amminikutty et al. (contribution 3), recognized as an Editor’s Choice article, showed that dietary turmeric powder counteracted oxidative stress and reduced hepatic aflatoxin B1 (AFB1) accumulation in broilers exposed to EU maximum permissible levels, underscoring the promise of phytogenic antioxidants as complementary detoxification tools. de Souza et al. (contribution 4) further broadened the mitigation toolbox by demonstrating that probiotic, paraprobiotic, and postbiotic *Lactobacillus*-based supplementation partially protected broiler intestinal integrity against the combined challenge of DON and *Clostridium perfringens*, a scenario that closely mirrors field conditions in which mycotoxicosis and enteric disease frequently coincide.

A second cluster of articles addressed the pathophysiology of mycotoxin exposure at the organ and systems level. Cui et al. (contribution 5) provided novel mechanistic insight by showing that ablation of gut microbiota alleviated DON-induced neurobehavioral abnormalities and brain damage in a murine model, implicating the gut–brain axis in the neurotoxic effects of trichothecenes—a relatively underexplored dimension of mycotoxicology with direct relevance to production animals. Kumar et al. (contribution 6) documented a field outbreak of aflatoxicosis in a herd of 524 water buffaloes in which 38 animals died over a six-month period, with the highest incidence among calves aged 6–12 months; affected animals also showed a 91.66% increase in abortion rate relative to the previous year, together with significant alterations in packed cell volume and serum bilirubin. Full clinical recovery was achieved within one month under a combined therapeutic and nutritional management protocol, providing clinically actionable guidance that is often absent from experimentally induced models. Shanmugasundaram et al. (contribution 7) examined the effects of subclinical, combined exposure to fumonisins, DON, and ZEN in broilers, showing dose-dependent impairment of immune response, amino acid digestibility, and intestinal morphology even at exposure levels below individual regulatory thresholds—a finding with direct implications for how regulatory limits set for single mycotoxins may underestimate real-world risk.

Finally, Rangel-Muñoz et al. (contribution 8) contributed an ecological and epidemiological perspective, demonstrating that houseflies act as vectors for aflatoxin- and zearalenone-producing *Aspergillus* and *Fusarium* species on dairy farms: across ten farms sampled in both dry and wet seasons, fly captures increased more than fivefold and the proportion of fungus-positive flies rose from 8.6% to 29.6% during the wet season, with a corresponding increase in the share of total mixed ration and raw milk samples exceeding regulatory aflatoxin and zearalenone limits. This article is a valuable reminder that mycotoxin risk management cannot be confined to feed formulation alone but must also account for on-farm biological and environmental vectors of contamination.

## 2. Recent Developments and Persisting Knowledge Gaps

The articles collected here should be read against a rapidly evolving broader literature. Climate change is increasingly recognized as a structural driver of mycotoxin risk: rising temperatures and shifting precipitation patterns are expanding the geographic range and pathogenicity of thermophilic and thermotolerant fungal species such as *Aspergillus flavus* and *Fusarium graminearum*, with cascading effects on the type, concentration, and co-occurrence of mycotoxins in feed crops [5]. Consistent with this, worldwide occurrence surveys have long documented mycotoxin positivity in the large majority of feed samples [2], and a recent national-scale survey found co-contamination rates for aflatoxin B1, deoxynivalenol, and zearalenone reaching roughly 60–100% across raw feed ingredients and complete feed products, with combinations such as DON + ZEN or AFB1 + DON + ZEN being particularly frequent [3]. On the analytical side, detection capabilities continue to advance rapidly, aided by steadily improving analytical sensitivity [1]: LC-MS/MS-based multi-mycotoxin panels are now complemented by lateral flow immunoassays, hyperspectral imaging, and machine-learning-integrated biosensors that promise faster, field-deployable screening. These analytical advances are mirrored in the recent literature focusing specifically on so-called emerging and masked mycotoxins—including modified *Fusarium* metabolites, *Alternaria* toxins, and other non-regulated analogs—whose occurrence, biosynthesis, toxicity, and control measures are increasingly well characterized [6], and in parallel reviews evaluating how portable, sensor-based platforms can bridge the gap between laboratory-grade chromatographic methods and the practical demands of on-farm feed screening [7].

The regulatory landscape further underscores the practical stakes of this research. Aflatoxin B1 levels in feed are subject to legally binding maximum limits under Directive 2002/32/EC on undesirable substances in animal feed [8], which sets markedly stricter thresholds for feed intended for dairy cattle, dairy sheep and goats, piglets, and young poultry than for general compound feed—precisely the production categories most heavily represented among this Special Issue’s contributions. For deoxynivalenol, zearalenone, ochratoxin A, T-2/HT-2 toxins, and fumonisins, Commission Recommendation 2006/576/EC [9] provides non-binding guidance values that Member States and feed producers are encouraged, though not legally required, to observe. This asymmetry between binding aflatoxin limits and advisory limits for other major mycotoxins is itself a source of regulatory complexity that the multicomponent detoxification strategies evaluated in this Special Issue (contributions 1, 2, 3, and 4) are well positioned to help address in practice.

Building on the momentum generated by this Special Issue, the author’s group has since extended this line of multicomponent-detoxification research to additional production stages and species—including weaned piglets and dairy sheep—with the accumulated evidence recently synthesized within a circular-economy and life-cycle-assessment framework [10]; these later studies are not themselves part of the Special Issue.

Comprehensive syntheses of mycotoxin exposure biomarkers across both human and animal matrices [11] point to a further opportunity: translating this harmonized biomarker and toxicokinetic knowledge into standardized, field-deployable monitoring tools for farm-animal production settings.

Viewed in this context, the Special Issue makes a timely contribution to at least three persisting knowledge gaps. First, several contributions (Raj et al., contribution 2; Shanmugasundaram et al., contribution 7; de Souza et al., contribution 4) deliberately modeled combined or subclinical mycotoxin exposures rather than single-toxin challenges, directly addressing the disconnect between regulatory frameworks—still largely built around single-mycotoxin thresholds—and the multi-mycotoxin reality documented in current surveillance data. Second, the demonstration by Cui et al. (contribution 5) that gut microbiota mediates DON-induced neurobehavioral and neuropathological outcomes opens a mechanistic avenue—the gut–brain–immune axis—that remains markedly underexplored in farm animal mycotoxicology relative to hepatic, renal, and reproductive endpoints. Third, the field study by Rangel-Muñoz et al. (contribution 8) and the clinical case series by Kumar et al. (contribution 6) help bridge the persistent gap between controlled experimental models and the more complex, multifactorial reality of mycotoxicosis as it presents on commercial farms, where vectors, husbandry practices, and concurrent disease interact with toxin exposure.

## 3. Future Research Directions

Building on the momentum of this Special Issue, several directions merit particular attention. Standardized, co-exposure experimental designs that reflect field-realistic mycotoxin combinations—rather than single-toxin challenges—should become the norm, supported where possible by dose–response and interaction (additive, synergistic, or antagonistic) modeling. The gut–brain–immune axis identified by Cui et al. (contribution 5) deserves systematic follow-up across production species, particularly given its potential relevance to behavior, welfare, and feed efficiency. Mechanistic and efficacy data on novel, non-adsorbent mitigation strategies—phytogenic antioxidants, probiotics, paraprobiotics, and postbiotics—should continue to be extended across production species, life stages, and husbandry systems, as illustrated by the recent progression from sows (contribution 1) to subsequent applications in weaned piglets and dairy sheep [10], and validated under commercial, rather than purely experimental, conditions, with attention to cost-effectiveness and regulatory approval pathways. Given the scale of the economic losses documented across the food and feed value chain [4], future work should also prioritize formal cost-effectiveness and return-on-investment analyses of detoxification strategies, to support producer and policymaker decision-making alongside the biological and toxicological evidence generated here. Given the demonstrated role of environmental vectors such as houseflies, integrated on-farm biosecurity and vector-control strategies deserve greater emphasis alongside feed-based interventions. The emerging circular-economy and life-cycle-assessment framework [10] should be extended to other species and detoxification strategies, formally linking mycotoxin mitigation to greenhouse gas, eutrophication, and acidification metrics within a One Health perspective. Portable, sensor-based detection platforms [7] and standardized, harmonized biomarker frameworks [11] should be adapted and validated for on-farm veterinary use, alongside systematic characterization of emerging and masked mycotoxins [6] in feed and animal-derived products. Finally, the accelerating pace of climate-driven shifts in mycotoxin occurrence argues for predictive, climate-informed risk modeling and for biomarker-based, on-farm monitoring tools capable of detecting real-world multi-mycotoxin exposure before it manifests as clinical disease.

## 4. Concluding Remarks

Collectively, these eight articles reinforce the view that effective mycotoxin risk management in farm animals requires an integrated strategy—combining improved detection, mechanistic understanding, multicomponent detoxification, and on-farm ecological awareness—rather than reliance on any single intervention. This Special Issue is expected to stand as a valuable and lasting reference for researchers, veterinarians, and the animal feed industry, and is anticipated to continue stimulating investigation into the many questions that remain open in this field.

## Figures and Tables

**Table 1:** Overview of the eight Special Issue contributions.

No.	Contribution and Species	Intervention/Model	Key Finding
1	Papatsiros et al.—sows	Multicomponent mycotoxin-detoxifying agent	Reduced oxidative stress markers; improved health and performance
2	Raj et al.—weaned pigs	Multicomponent detoxifying agent vs. combined DON + ZEN challenge	Improved health and performance under combined dietary exposure
3	Amminikutty et al.—broilers (Editor’s Choice)	Turmeric powder vs. AFB1 at EU maximum feed levels	Reduced hepatic AFB1 content; counteracted oxidative stress
4	de Souza et al.—broilers	Probiotic/paraprobiotic/postbiotic vs. DON + *Clostridium perfringens*	Modulated intestinal changes under combined mycotoxin–enteric challenge
5	Cui et al.—mice	Gut microbiota ablation vs. DON exposure	Alleviated DON-induced neurobehavioral abnormalities and brain damage
6	Kumar et al.—water buffalo	Field outbreak of aflatoxicosis (herd of 524)	38 deaths; 91.66% rise in abortion rate; full recovery within one month under combined protocol
7	Shanmugasundaram et al.—broilers	Subclinical combined fumonisins + DON + ZEN	Dose-dependent impairment of immune response, digestibility, and intestinal morphology
8	Rangel-Muñoz et al.—dairy farms	Houseflies as vectors for *Aspergillus*/*Fusarium* spp.	>5-fold rise in fly captures; fungus-positive flies rose from 8.6% to 29.6% in wet season
